# Supplementary material for: Sugar Levels Determine Fermentation Dynamics during Yeast Pastry Making and Its Impact on Dough and Product Characteristics
Source: Foods. 2022 May 11;11(10):1388. doi: 10.3390/foods11101388 (PMC9140867; doi:10.3390/foods11101388)
Supplement: Supplementary file 1 [file foods-11-01388-s001.zip › foods-1680625-supplementary.pdf]

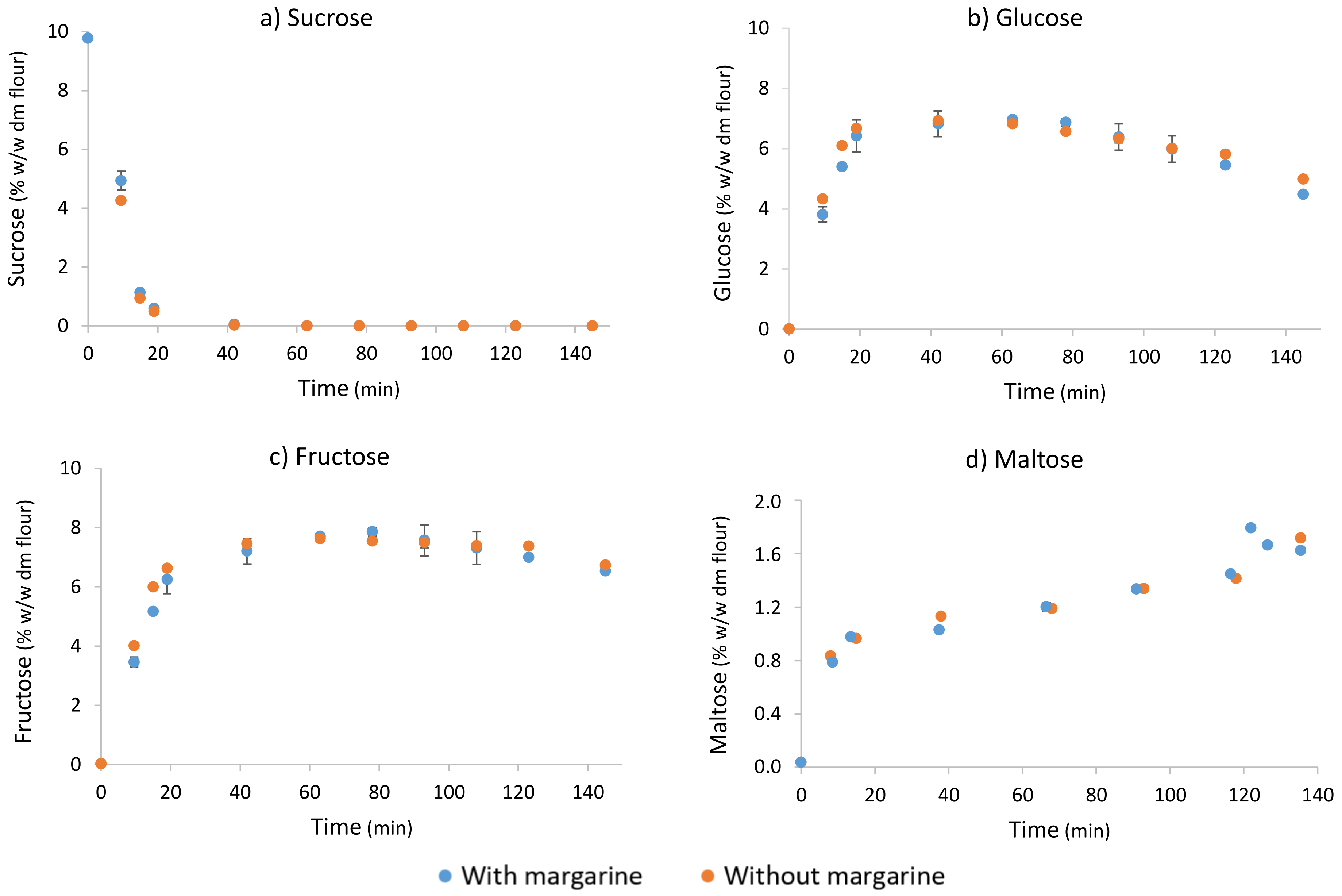


**Figure S1**: Evolution of (**a**) sucrose, (**b**) glucose, (**c**) fructose, and (**d**) maltose concentrations in pastry samples with 8% yeast and with or without margarine as a function of time (min) during pastry production. Concentrations are expressed on flour dry matter base (% w/w dm flour). The start of mixing is defined as t = 0. Vertical bars represent standard deviations of duplicate measurements.
